# Insecticide susceptibility of *Aedes aegypti* populations from Senegal and Cape Verde Archipelago

**DOI:** 10.1186/1756-3305-5-238

**Published:** 2012-10-22

**Authors:** Ibrahima Dia, Cheikh Tidiane Diagne, Yamar Ba, Diawo Diallo, Lassana Konate, Mawlouth Diallo

**Affiliations:** 1 Unité d’entomologie médicale, Institut Pasteur de Dakar, 36 Avenue Pasteur, Dakar BP 220, Sénégal; 2 Laboratoire d’écologie vectorielle et parasitaire, Département de Biologie Animale, Université Cheikh Anta Diop, Dakar BP 5005, Senegal

## Abstract

**Background:**

Two concomitant dengue 3 (DEN-3) epidemics occurred in Cape Verde Archipelago and Senegal between September and October 2009. *Aedes aegypti* was identified as the vector of these epidemics as several DEN-3 virus strains were isolated from this species in both countries. The susceptibility to pyrethroids, organochlorine, organophosphates and carbamate was investigated in two field strains of *Aedes aegypti* from both countries using WHO diagnostic bioassay kits in order to monitor their the current status of insecticide susceptibility.

**Findings:**

The two tested strains were highly resistant to DDT. The Cape Verde strain was found to be susceptible to all others tested insecticides except for propoxur 0.1%, which needs further investigation. The Dakar strain was susceptible to fenitrothion 1% and permethrin 0.75%, but displayed reduced susceptibility to deltamethrin, lambda-cyhalothrin and propoxur.

**Conclusions:**

As base-line results, our observations stress a careful management of insecticide use for the control of *Ae. aegypti*. Indeed, they indicate that DDT is no longer efficient for the control of *Ae*. *aegypti* populations in Cape Verde and Dakar and further suggest a thorough follow-up of propoxur susceptibility status in both sites and that of deltamethrin and lambda-cyhalothrin in *Ae. aegypti* populations in Dakar. Thus, regular monitoring of susceptibility is greatly needed as well as the knowing if this observed resistance/susceptibility is focal or not and for observed resistance, the use of biochemical methods is needed with detailed comparison of resistance levels over a large geographic area.

**Keywords:**

*Aedes aegypti*, Insecticides, Susceptibility, Cape Verde, Senegal

## Findings

### Background

Between September and October 2009, two concomitant epidemics of dengue caused by the serotype dengue 3 (DEN-3) were recorded in Senegal and Cape Verde Archipelago [1,2]. In Senegal 696 suspected cases were recorded with 196 confirmed while in Cape Verde, there were an estimated 210,000 clinical cases of which 174 fitted the WHO definition of DHF/DSS and six were fatal. The majority of cases were on Santiago, Fogo and Maio in the leeward (southern) chain of islands whereas in Senegal the cities of Dakar, Louga and Mbour were of concern. *Aedes aegypti* was identified as the vector of these epidemics as several DEN-3 strains were isolated from this species in both countries. In Cape Verde archipelago, since its first report in S. Vicente Island in 1931 [3], it was recently described in Santiago, Fogo and Brava islands [4,5]. In Senegal, the presence of *Ae. aegypti* is widespread as it was observed in all bioclimatic zones [6,7]. In response to these epidemics, the Ministry of Health of the two countries launched an emergency vector control campaign, including a major public education effort, and energetic source reduction operation, backed by treatment of breeding sites (mainly in Cape Verde archipelago by temephos use) and indoor and outdoor residual treatment with insecticidal aerosols*.* Respectively, deltamethrin and propoxur were the insecticides used without proper consideration to control adult *Aedes* mosquitoes through mass spraying while: i) no information was available on the status of *Ae. aegypti* populations to these insecticides and ii) the development of resistance to organochlorine, organophosphate, carbamate, and pyrethroid insecticides in *Ae. Aegypti* from subtropical and tropical regions of the world can hinder control efforts [8]. Indeed, as multiple resistances to pyrethroids and organophosphates has been reported in *Ae. aegypti* populations from Asia [9,10], South America and The Caribbean [11-14] and in a lesser extent in Africa [15], control efforts can seriously be compromised by such phenomenon. Therefore, the present work was undertaken to monitor the current status of insecticide susceptibility in *Ae. aegypti* populations collected from Senegal and Cape Verde for compounds that are in current or planned for future use.

### Methods

*Ae. aegypti* eggs, 3rd and 4th larvae stages were collected in Dakar (14°45’N, 17°27’W) and in Santiago island (14°54’N, 23°31’W) in November 2009. Pupae were collected and kept in cages until adult emergence. Adult mosquitoes were maintained with a 10% sucrose solution.

Bioassays were carried out using WHO tests kits for adult mosquitoes [16]. Insecticide-impregnated papers were provided by The Vector Control Research Unit, School of Biological Sciences (Universiti Sains Malaysia), a WHO Collaborating Centre. The following diagnostic concentrations of insecticides were tested: 4% DDT, 1% fenitrothion, 0.1% propoxur, 0.75% permethrin, 0.05% lambda-cyhalothrin, and 0.05% deltamethrin. For DDT and propoxur, the discriminating concentrations from WHO criteria were used whereas for the others, literature data from previous published studies were used [9,17-19].

Tests were performed on 2–5 day-old unfed females. Batches of 20–25 females were exposed to insecticide-impregnated papers for 30 min to 4% DDT and 1 hour to the other tested insecticides. The number of knockdown mosquitoes was recorded every 10 min during exposure. The knockdown times for 50% (KdT_50_) and 95% (KdT_95_) of tested mosquitoes were calculated using a log-probit software (StatPlus, version 2009) according to [20]. The mortality rate was recorded after 24 h. Tests with untreated papers were systematically run as control. When control mortality was between 5 and 20%, mortality rate in tested samples was corrected using Abbott formula [21]. The WHO criteria for determining resistance or susceptibility were applied [8].

### Results and discussion

Resistance to DDT was detected both in Cape Verde and Dakar strains, as mortality rates 24h post exposure obtained with this insecticide were respectively 33.3 and 24.4%. The Cape Verde strain was found to be susceptible to fenitrothion 1% and the three tested pyrethroids (deltamethrin 0.05%, lambda-cyhalothrin 0.05% and permethrin 0.75%). The mortality rate for propoxur was 87.2%, suggesting suspected resistance, and the need of further investigation.

By contrast, the Dakar strain was susceptible only to fenitrothion 1% and permethrin 0.75%. The mortality rates were respectively 87.2% for propoxur 1%, 94.5% for deltamethrin 0.05% and 81.6% for lambda-cyhalothrin 0.05% (Table 1). These results also suggest suspected resistance and need to be confirmed.

**Table 1:** **Percentage mortality in field samples of *Aedes aegypti *24 hours after exposure to insecticide-impregnated papers and correspondence with knockdown time (KdT) using WHO test tubes**

**Insecticides (diagnostic dose)**	**Cape Verde strain**					**Dakar strain**				
	**N**	**Mortality (%)**	**KDT**_**50**_**(min)**	**KDT**_**95**_**(min)**	**Resistance status**	**N**	**Mortality (%)**	**KDT**_**50**_**(min)**	**KDT**_**95**_**(min)**	**Resistance status**
DDT (4%)	231	33.3	78.8	288.7	R	214	24.4	-	-	R
Propoxur (0.1%)	233	87.2	23.5	38.8	RPC	228	87.2	23.9	73.9	RPC
Fenitrothion (1%)	123	100	-	-	S	125	99.1	-	-	S
Deltamethrin (0.05%)	228	100	9.6	27	S	189	94.5	15.7	30.3	RPC
Lambda-Cyhalothrin (0.05%)	125	100	14.5	26.9	S	125	81.6	18.6	34.2	RPC
Permethrin (0.75%)	123	100	7.2	21.8	S	125	100	11.3	20.5	S

N=number of specimens tested; S=susceptible: R=resistant; RPC=resistance possible to be confirmed.

For the two strains tested, the relationship between knockdown time and the time for exposure was linear. No knock down effect was observed with DDT 4% for the Dakar strain, for which KdT_50_ and KdT_95_ were higher in comparison with the other tested insecticides.

For Permethrin 0.75%, KdT_50_ and KdT_95_ were comparable between the two strains while, for the remaining pyrethroids, KdT_50_ and KdT_95_ were higher for Dakar strain in comparison with Cape Verde strain (Table 1).

According to these results, the Cape Verde and Dakar strains tested are resistant to DDT 4% and exhibit a reduced susceptibility to propoxur 1%. Similar results on DDT resistance were recently obtained in Central Africa in areas where *Ae. aegypti* and *Ae. albopictus* are sympatric [15] . This observation is not surprising since DDT and propoxur are extensively used in these areas for mosquito control as well as for market gardening crop protection causing contamination of natural breeding sites [22]. For DDT, these authors have shown huge resistance of the main malaria vector (*Anopheles arabiensis*) in Dakar and Pikine, one of the main surrounding neighbourhoods. Indeed, the contamination of larval breeding sites by insecticide used in agriculture is often associated to the selection for DDT and others insecticides resistance in diseases vectors [23,24]. Residual spraying using DDT was the principal method for malaria eradication in Cape Verde archipelago [25] and *Ae. aegypti* control in Dakar [26] between the 1940s and 1960s. As a result, the long term and extensive use of DDT would have led to a strong selective pressure on exposed mosquito populations and could explain the high resistance to DDT observed in our two strains. Indeed, DDT resistance has been widely observed in *Ae. aegypti* worldwide. *Kdr* mutation of the voltage-gated sodium channel as well as detoxifying enzymes such as glutathione S-transferases have been found to play a key role in DDT metabolism and resistance [27-29]. Therefore, as cross-resistance between DDT and pyrethroids has been extensively demonstrated to be associated with *kdr* mutation in their common target site, this may have serious consequences for insecticide-based *Ae. aegypti* control programmes. For the other tested insecticides, the susceptibility of Cape Verde strain compared to the Dakar strain could be the result of non-use or rare use of these insecticides. In fact, apart from deltamethrin that is extensively used in IRS for malaria control, no other official insecticide is used for vector control.

For the Dakar strain, there is a decreased susceptibility to deltamethrin 0.05% and lambda-cyhalothrin 0.05%. However, it is interesting to note that the KDT_50_ and KDT_95_ are comparable with the values obtained with the Cape Verde strain that was fully susceptible to these insecticides.

### Conclusions

This study reports for the first time the susceptibility of *Ae. aegypti* populations to commonly used insecticides. Our results indicate that DDT is no longer efficient for the control of *Ae*. *aegypti* populations in Cape Verde and Dakar. They further suggest a thorough follow-up of propoxur susceptibility status in both sites and that of deltamethrin and lambda-cyhalothrin in *Ae. aegypti* populations in Dakar. In addition, regular monitoring of susceptibility is greatly needed. It is also important to know if this observed resistance/susceptibility is focal or not and for observed resistance, the use of biochemical methods is needed with detailed comparison of resistance levels over a large geographic area.

## Competing interests

The authors declare that they have no competing interests.

## Authors’ contributions

ID and MD designed and supervised the study. ID, CTD and DD performed the susceptibility tests. ID, CTD, MD analyzed the data. ID and MD drafted and revised the manuscript. All authors revised and approved the final version of the manuscript.
